# SARS-CoV-2: The Second Wave in Europe

**DOI:** 10.2196/22431

**Published:** 2021-05-18

**Authors:** Athanassios S Fokas, George A Kastis

**Affiliations:** 1 Department of Applied Mathematics and Theoretical Physics University of Cambridge Cambridge United Kingdom; 2 Viterbi School of Engineering University of South California Los Angeles, CA United States; 3 Mathematics Research Center Academy of Athens Athens Greece

**Keywords:** mathematical modelling of epidemics, COVID-19, SARS CoV-2, pandemic, lockdown in Europe

## Abstract

Although the SARS-CoV-2 virus has already undergone several mutations, the impact of these mutations on its infectivity and virulence remains controversial. In this viewpoint, we present arguments suggesting that SARS-CoV-2 mutants responsible for the second wave have less virulence but much higher infectivity. This suggestion is based on the results of the forecasting and mechanistic models developed by our study group. In particular, in May 2020, the analysis of our mechanistic model predicted that the easing of lockdown measures will lead to a dramatic second wave of the COVID-19 outbreak. However, after the lockdown was lifted in many European countries, the resulting number of reported infected cases and especially the number of deaths remained low for approximately two months. This raised the false hope that a substantial second wave will be avoided and that the COVID-19 epidemic in these European countries was nearing an end. Unfortunately, since the first week of August 2020, the number of reported infected cases increased dramatically. Furthermore, this was accompanied by an increasingly large number of deaths. The rate of reported infected cases in the second wave was much higher than that in the first wave, whereas the rate of deaths was lower. This trend is consistent with higher infectivity and lower virulence. Even if the mutated form of SARS-CoV-2 is less virulent, the very high number of reported infected cases implies that a large number of people will perish.

## Introduction

The novel coronavirus SARS-CoV-2 has already undergone several mutations. A study claimed that a specific mutation had created a more “aggressive” form of the virus [1]. However, it was later argued that, during epidemics in general, a virus spreads to new areas and creates new localized epidemics that grow exponentially [2]. This finding implies that a mutated virus spreading in these new uninfected areas will rapidly increase its mutation frequency; this will occur even if the mutation is neutral or detrimental to the virus itself. This study also showed that the observations reported by Tang et al [1] could be explained by the abovementioned process and that there was *no* evidence for the emergence of a more aggressive form of the virus [2]. Another study analyzed a set of mutations in the spike protein of SARS-CoV-2 and concluded that a specific mutation had enhanced the capacity of the virus to spread from China to Europe, North America, and Australia [3]. These authors concluded that this was the result of the fact that this mutation had made the virus more “transmissible,” allowing it to outcompete other virus strains that did not possess this mutation. However, this conclusion was challenged by several scientists. In particular, a more formal analysis of the effect of this mutation by another research team suggested that it actually has reduced transmissibility [4]. It has now been firmly established that the second wave was caused by a new mutated form of SARS-CoV-2, called D614G—the name refers to the substitution of the amino acid aspartic acid (D) by glycine (G) in a region of the viral genome that encodes the relevant spike protein. In particular, a study conducted in Houston, Texas, United States, found that D614G is the fully dominant form of the second wave of the COVID-19 pandemic, exhibiting a prevalence of 99.9% [5]. The study also found that people infected with the new SARS-CoV-2 strain had higher loads of the virus in their upper respiratory tracts, which suggests that the D614G strain may be more infectious; however, no definitive conclusion regarding its infectivity has been reached. The high prevalence of the new strain was also established in a study in the United Kingdom involving the analysis of 25,000 whole-genome SARS-CoV-2 sequences [6], but no conclusions were reached regarding its infectivity or virulence. Therefore, the crucial question of establishing whether the new strain D614G was indeed more infectious and/or less virulent remained open [7].

In the framework introduced by Holmdahl and Buckee [8], epidemiological models are broadly divided into two categories: *forecasting* and *mechanistic*. The former models fit a specific curve to the data and then attempt to predict the dynamics of the quantity under consideration. The main limitation of these models is that they remain valid only if the epidemiological situation remains unchanged. For example, they can be used during a lockdown period but will not make accurate predictions after the lockdown is lifted. In contrast to forecasting models, mechanistic models can make predictions even when the relevant circumstances change. Their main limitation is the difficulty of determining the parameters specifying such models.

Our group has developed both forecasting and mechanistic models. In this paper, we present the implications of our models to argue that the mutated virus D614G was more infectious but less virulent.

## The Prediction of the Effect of Easing Lockdown Measures by Using a Mechanistic Type Model

The generic weakness of mechanistic models, namely the difficulty in determining the parameters of the given model, was partially bypassed in a previous study [9]. Indeed, this study showed that the death data *uniquely* determine the parameters specifying a given ordinary differential equation characterizing the number of deaths. Furthermore, a robust numerical algorithm was presented for obtaining these parameters. The abovementioned equation was obtained from the manipulation of a susceptible-exposed-infected-recovered (SEIR) type model. Using this methodology, predictions were made about the dynamics of the COVID-19 epidemics in Greece, Andalusia, and Portugal, following the easing of lockdown measures. One of the above parameters obtained from the death data during the lockdown period corresponds to the number of contacts of the asymptomatic individuals. By changing this parameter (doubling, tripling, etc), while keeping all other parameters unchanged, the effect of relaxing the lockdown measures could be *quantified*: if the contacts of the asymptomatic people double, nothing much happens; but if they triple, then the situation dramatically deteriorates. The numbers of reported infected cases during the second wave was within the range of our prediction made in May 2020; fortunately, the number of deaths reported was lower, suggesting less virulence.

## Cumulative Number of Deaths and Cumulative Number of Reported Infected Cases

It is correctly noted in a previous study [10] that the forecasting models are “not well suited for long-term predictions.” However, we have introduced a forecasting model that *can* provide *highly accurate long-term predictions* both for the number of deaths caused by COVID-19 and the number of reported infected cases. Our success was based on replacing the usual logistic formula used in epidemiology by slightly more general formulas, namely, what we have called rational and birational formulas. Interestingly, these formulas are, in a sense, optimal, since elaborate “deep learning” algorithms could *not* improve the predictions obtained by using these formulas [11].

**Figure 1:**
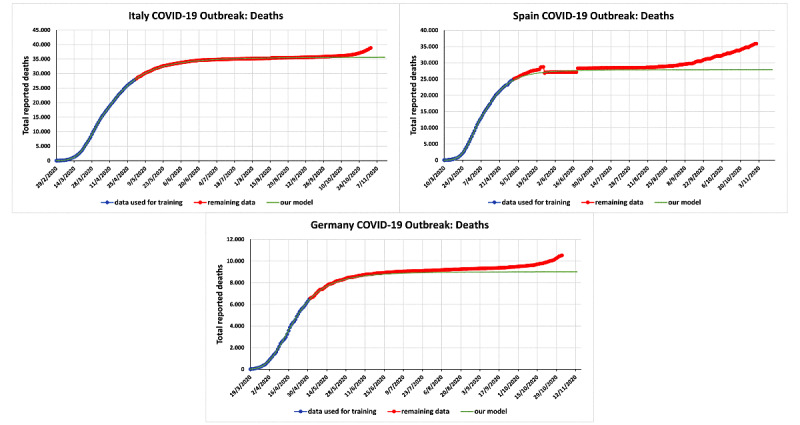
Actual versus predicted cumulative number of reported deaths due to COVID-19 for Italy, Spain, and Germany. The constants of our mathematical model were determined using data up to May 1, 2020, which are depicted in light blue. The red curves correspond to the actual data up to November 2, 2020, which are the data during the period of our predictions. Our predictions are depicted in green.

By training a formula to use data for the accumulative number of deaths in a given country until May 1, 2020, we were able to make predictions that were valid well beyond the end of the first wave, namely, for a period of more than 3.5 months. As shown in Figure 1, in Italy, there was no deviation between the curve depicting the number of deaths and the curve of our predictions, whereas in Germany, a small deviation began to occur in the second week of August 2020. In Spain, a larger deviation began to occur in the first week of August 2020 [12]. Our predictions for the number of reported infected cases were equally accurate [11].

These graphs show that following the easing of the first lockdown, the number of deaths did not increase in several European countries for approximately two months. Figure 1 presents three graphs depicting the total number of deaths caused by the COVID-19 epidemics in Italy, Spain, and Germany. These graphs compare the red curves indicating *real data* from the period of May 2 to November 2, 2020, with the green curves indicating the *predictions* made with an explicit mathematical formula introduced previously [11]. This formula contains 4 explicit constants that were determined by using data up to May 1, 2020. The green curves depicting this formula are indistinguishable from the red curves depicting the real data until mid-August 2020, despite the fact that the lockdown was lifted in the above countries by mid-June 2020.

**Figure 2:**
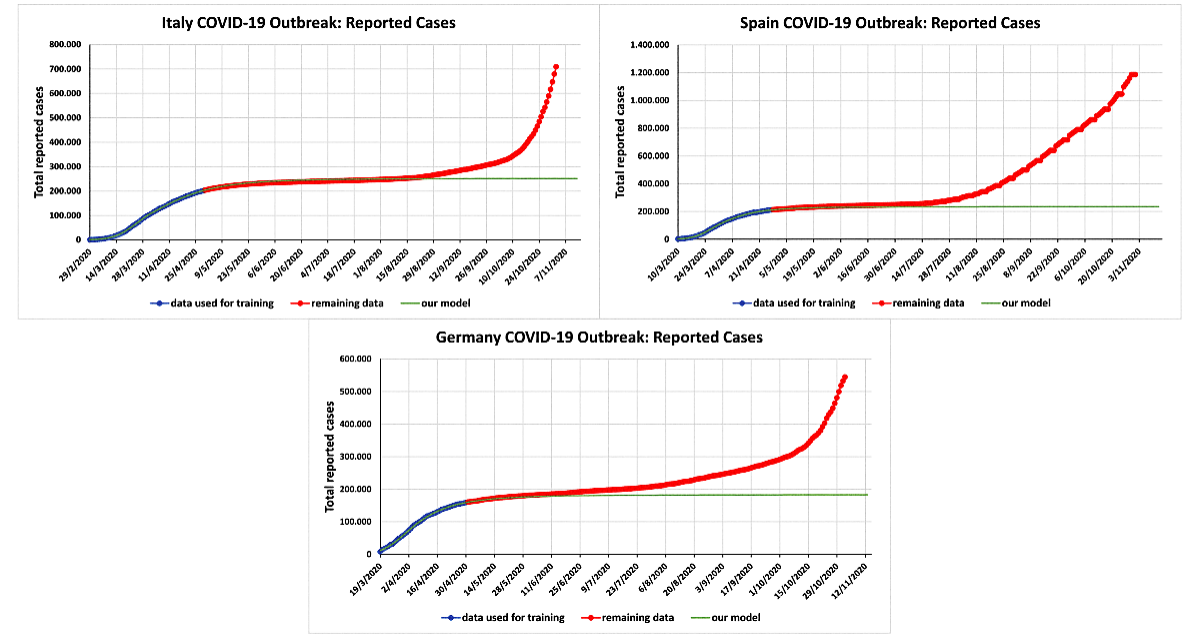
Actual versus predicted cumulative numbers of reported infected individuals with COVID-19 in Italy, Spain, and Germany. The prediction fits were obtained using data up to April 29, 2020, for Italy; April 27, 2020, for Spain; and April 30, 2020, for Germany. The relevant constants were determined with the data shown in light blue. The red curves correspond to the actual data up to November 2, 2020, which are the data during the period of our predictions. Our predictions are depicted in green.

Figure 2 presents analogous curves for the number of reported infected cases. Comparing the red curves depicting the real data with the green curves depicting our predictions, it follows that until August-end for Italy, mid-July for Spain, and June-end for Germany, in 2020, the effect of the elimination of several restrictive measures had only a slight effect on the numbers of reported infected cases (compared with the numbers that would have occurred during the lockdown).

## Estimates for the Number of Reported Infected Cases and Deaths in Israel

We have applied our formulas to the cumulative number of deaths and reported infected cases for the third wave of COVID-19 in Israel. For the number of deaths, the rational formula predicted a plateau on April 8, 2021, with 3234 deaths and a total of 6060 deaths overall. The plateau is defined *as the point at which* *the rate of deaths for the given wave is 5% of the maximum rate.* For the number of reported infected cases in Israel, the rational formula predicted a plateau on April 16, 2021, with 497,992 cases for the third wave and a total of 825,399 cases overall. In Israel, the rate of infections of the third wave reached a maximum on January 20, 2021.

## Higher Infectivity and Less Virulence

The predictions reported previously [13] were based on the assumption that *the characteristics of the virus remained unchanged.* The initial period following the easing of lockdown measures, when the number of deaths in the abovementioned countries did *not* increase and the number of reported infected cases was small, provided hope that the virulence and infectivity of the mutated form of the virus was much lower. However, the subsequent increase in the number of confirmed cases and deaths suggests an alternative explanation: the results reported previously [13] showed that if the easing of the restrictive measures leads up to doubling of the contacts of asymptomatic persons, the situation still remains essentially the same as it was during the lockdown period. Moreover, apparently, immediately after the lockdown period, the population at large continued to observe social distancing and other protective measures. Unfortunately, following this period of high vigilance, the population, especially younger individuals, thought that the danger was over and began enjoying the summer without observing the necessary protective measures.

A possible explanation for the observation that the number of deaths in the second wave was lower than the number expected given the very high number of infected individuals includes a number of factors. First, better medical practices and treatment are available. Second, older individuals and those with comorbidities are careful and avoid social contact. Indeed, the SARS-CoV-2 infection in younger individuals rarely leads to death. By extending the algorithm developed [13] into two subpopulations consisting of younger (below 40 years) and older (above 40 years) individuals, a previous study [9] on the epidemic in Greece found that if the number of contacts between asymptomatic “younger” persons increases, the number of reported infected cases increases but the number of deaths does not. In contrast, an increase in the number of contacts involving “older” persons leads to a dramatic increase in the number of deaths. Third and most importantly, it is possible that the mutated virus D614G was less virulent.

By directly computing the rate of change in the number of deaths in the second wave by using real data, it becomes clear that this rate is lower than the rate in the first wave, thereby supporting the suggestion that the mutated virus is less virulent. However, analogous computations for the rate of the change of the number of reported infected shows that this rate is much higher for the second wave, suggesting a much higher infectivity.

Concluding, it is worth noting that the period immediately following the easing of lockdown measures provides a very useful lesson. By using the well-publicized measures of social distancing, environmental hygiene, hand washing, and appropriate use of masks, it is possible to control the COVID-19 epidemic without imposing a strict lockdown.

## Abbreviations

SEIR: susceptible-exposed-infected-recovered
